# The effect of diabetes mellitus on perioperative outcomes after colorectal resection: a national cohort study

**DOI:** 10.1016/j.bja.2024.04.010

**Published:** 2024-05-16

**Authors:** Savannah Gysling, Christopher A. Lewis-Lloyd, Dileep N. Lobo, Colin J. Crooks, David J. Humes

**Affiliations:** 1 Nottingham Digestive Diseases Centre, Division of Translation Medical Sciences, School of Medicine, University of Nottingham, Queen's Medical Centre, Nottingham, UK; 2 National Institute for Health Research (NIHR) Nottingham Biomedical Research Centre, Nottingham University Hospitals NHS Trust and University of Nottingham, Queen's Medical Centre, Nottingham, UK; 3 MRC Versus Arthritis Centre for Musculoskeletal Ageing Research, School of Life Sciences, University of Nottingham, Queen's Medical Centre, Nottingham, UK; 4 Division of Surgery, Perelman School of Medicine, University of Pennsylvania, Philadelphia, PA, USA

**Keywords:** cohort study, colorectal surgery, complications, diabetes mellitus, insulin, mortality, outcomes

## Abstract

**Background:**

Diabetes mellitus is a significant modulator of postoperative outcomes and is an important risk factor in the patient selection process. We aimed to investigate the effect of diabetes mellitus and use of insulin on outcomes after colorectal resection using a national cohort.

**Methods:**

Adults with a recorded colorectal resection in England between 2010 and 2020 were identified from Hospital Episode Statistics data linked to the Clinical Practice Research Database. The primary outcome was 90-day mortality. Secondary outcomes included hospital length of stay (LOS) and readmission within 90 days.

**Results:**

Of the 106 139 (52 875, 49.8% male) patients included, diabetes mellitus was prevalent in 10 931 (10.3%), 2145 (19.6%) of whom had a record of use of insulin. Unadjusted 90-day mortality risk was 5.7%, with an increased adjusted hazard ratio (aHR) for people with diabetes mellitus (aHR 1.28, 95% confidence interval [CI] 1.19–1.37, *P*<0.001). This risk was higher in both people with diabetes using insulin (aHR 1.51, 95% CI 1.31–1.74, *P*<0.001) and not using insulin (aHR 1.22, 95% CI 1.13–1.33, *P*<0.001), compared with those without diabetes. Ninety-day readmission occurred in 20 542 (19.4%) patients and this was more likely in those with diabetes mellitus (aHR 1.23, 95% CI 1.18–1.29, *P*<0.001). Median (inter-quartile range) LOS was 8 (5–15) days and was higher in people with diabetes mellitus (adjusted time ratio 1.10, 95% CI 1.08–1.11, *P*<0.001).

**Conclusions:**

People with diabetes mellitus undergoing colorectal resection are at a higher risk of 90-day mortality, prolonged LOS, and 90-day readmission, with use of insulin associated with additional risk.

**Editor's key points**

• Diabetes mellitus is an important risk factor for poor postoperative outcomes.
• Diabetes care is evolving rapidly with new treatments which could modify perioperative risk.
• These UK National Health Service data show that patients with diabetes mellitus continue to be exposed to additional perioperative risk.
• Further research is needed to monitor and tackle the evolving risks for diabetic patients undergoing major surgery.

Diabetes mellitus affects >9% of the global population, with an increasing prevalence recorded over the last 15 yr.^1^, ^2^, ^3^ People with diabetes mellitus make up 10–14% of all patients undergoing surgery,^4^ and 12–14% of those having colorectal surgery.^5^^,^^6^ There is also a growing awareness of people with undiagnosed diabetes undergoing surgery, with an estimated prevalence of 7.4%.^7^

There are several mechanisms through which people with diabetes mellitus might potentially experience delayed postoperative recovery and poorer outcomes, including an increased comorbidity burden resulting from complications of diabetes, errors in insulin prescribing, and perioperative management of medicines for diabetes, episodes of hypo- and hyperglycaemia, dysregulated inflammatory response to surgery, and impaired wound healing.^8^, ^9^, ^10^, ^11^

Findings derived from a range of surgical specialties have led to a general understanding of diabetes mellitus as a significant modulator of postoperative outcomes, and as an important risk factor when evaluating the appropriateness of surgery for each individual. As a result, several national and international guidelines on the perioperative optimisation and risk modification of people with diabetes undergoing surgery have been published,^9^^,^^12^, ^13^, ^14^, ^15^ although adherence to these is often poor.^8^^,^^16^

Despite this general understanding of diabetes mellitus as a risk factor for adverse perioperative outcomes, there is an overall paucity of evidence describing the impact of diabetes on postoperative outcomes in people undergoing colorectal surgery. Moreover, within the field of colorectal surgery, there is a lack of large-scale studies to specifically quantify any additional risk that diabetes may confer, to aid in clinical and shared decision-making.

The literature describing the impact of diabetes in people undergoing colorectal resections has recently been summarised in a meta-analysis,^5^ which reported no statistically significant difference in 30-day mortality between people with and without diabetes after colorectal surgery (odds ratio [OR] 2.65, 95% confidence interval [CI] 0.80–8.70, *P*=0.109). This imprecision reflects the limited evidence that could be pooled for analysis of this outcome; with only three studies encompassing just 3526 patients with diabetes.

Lastly, there is limited information on whether preoperative use of insulin, considered here as a marker for disease severity, results in any change in the risk of poorer postoperative outcomes in people with diabetes mellitus who present for colorectal resection, in comparison with those with diabetes without preoperative use of insulin and those without diabetes. Any potential effect of the use of insulin on postoperative outcomes in people undergoing colorectal surgery remains to be quantified.

We aimed to investigate and quantify the effect of diabetes mellitus and preoperative use of insulin on 90-day mortality, postoperative hospital length of stay (LOS), and 90-day readmission rates after colorectal resections using a national cohort from England.

## Methods

### Study design and ethical approval

This national, observational cohort study was designed to compare outcomes for people with and without diabetes, and with and without insulin use, undergoing colorectal surgery. The study was performed and reported in accordance with the Strengthening the Reporting of Observational Studies in Epidemiology (STROBE) guidelines.^17^ Ethical approval and permission to use the Clinical Practice Research Datalink (CPRD)^18^ were obtained from the Independent Scientific Advisory Committee (ISAC) approval board (protocol number 21_000404).

### Setting and participants

All patients aged 16 yr and over who underwent surgery for colorectal disease in England over a 20-yr period (January 1, 2000 to December 31, 2019) were identified within the Hospital Episode Statistics (HES) database using Office of Population, Censuses and Surveys Classification of Surgical Operations and Procedures Version 4 (OPCS-4) codes.^19^ Malignant and benign indications for colorectal surgery were included, with colorectal resections for a malignant indication differentiated from those with a benign indication (inflammatory bowel disease, diverticular disease or other) by use of *International Classification of Diseases and Related Health Problems* 10th *Revision* (ICD-10) codes,^20^ including C18, C19, and C20 for colorectal malignancy. Patients were additionally classified as having an operation for a malignancy if a diagnosis of colorectal cancer was recorded in the CPRD within 6 months of surgery. Colorectal surgery of any type was included, with operative details regarding surgical approach (open *vs* laparoscopic *vs* robotic) and urgency (elective *vs* emergency) obtained from HES data. All codes are listed in Supplementary Table S1.

### Exposure variables

Using CPRD Read codes (Supplementary Table S1), data on the preoperative diabetes status (yes or no), type of diabetes (type 1 *vs* type 2) and any use of insulin preceding the date of surgery (yes or no) were extracted from the CPRD. Gestational and type 3c diabetes subtypes were excluded. Similarly, those with type 1 diabetes who did not have a record of insulin use were excluded.

Comorbidity burden, as classified before the hospital admission according to the Charlson Comorbidity Index (CCI),^21^ was obtained from CPRD and HES data. Patients were grouped according to a CCI score of zero (CCI 0), one (CCI 1), or two or more (CCI 2). Diabetes was excluded as a contributor to the CCI score, to prevent co-linearity and allow for an independent analysis of this variable. Information on relative deprivation was similarly retrieved from the CPRD, in the form of Index of Multiple Deprivation 2015 scores,^22^ ranging from least deprived (first quintile) to most deprived (fifth quintile).

### Data sources

Linked patient data for any adult undergoing a first colorectal resection in England between January 1, 2000 and December 31, 2019 were extracted from the CPRD Aurum, HES, and Office for National Statistics (ONS) databases. This represents prescription and diagnostic data from a national primary care database (CPRD), and information on each hospital admission within the National Health Service (NHS), captured by a national secondary care database (HES). Patients are linked across databases by a third party.

The CPRD provides an ‘acceptable for research’ flag to assess the reliability, validity, and continuity of data for use in research, and adherence of primary care practices to the minimum quality criteria determined by the CPRD.^18^ Any data that did not meet these quality assurance criteria were excluded.

### Study outcomes

The primary outcome was 90-day mortality, defined as all-cause mortality within 90 days of the index operation. Secondary outcomes included 90-day readmission, defined as any emergency readmission within 90 days of discharge, and hospital LOS, defined as days spent in hospital from the date of hospital admission to the date of discharge. Data pertaining to these outcomes were obtained from ONS death certificate records (mortality status), the HES database (in-hospital mortality, hospital LOS, 90-day readmission), and further validated using the CPRD (mortality status).

### Statistical analysis

Continuous variables were summarised using mean and standard deviation (sd) or median and inter-quartile range (IQR). Effect sizes were compared between groups using unpaired *t*-tests for parametric data, and Wilcoxon Rank Sum tests for non-parametric data. Categorical variables were presented as frequency and percentage, and compared using χ^2^ tests, with results presented as OR. Missing data pertaining to exposure variables were presented separately.

Kaplan–Meier survival functions and Kaplan–Meier failure functions were used to describe 90-day mortality and 90-day readmission for patients with or without the exposure of interest (diabetes *vs* no diabetes, type 1 *vs* type 2 diabetes, and use of insulin *vs* no use of insulin). The difference in survival functions between groups was tested using log rank tests. Time at risk was totalled to calculate crude 90-day mortality rates per person-time.

Adjusted mortality rates were modelled using Cox proportional hazards regression, accounting for the covariates: age, sex, deprivation, comorbidity burden, diabetes status, operation urgency, operation indication, and surgical access (Supplementary Table S2). Each exposure variable was investigated using univariable analysis based on an available case approach, with those reaching statistical significance included in the final multivariable Cox regression model. Likelihood ratio tests were used to determine the overall significance of factor variables for inclusion in the final model. Results from the final model were presented as adjusted hazard ratios (aHR).

An additional Cox regression model was developed to assess the impact of use of insulin on 90-day mortality using a similar process. This model compared people with diabetes with use of insulin, those with diabetes without use of insulin, and those without diabetes as a reference group. Ninety-day readmission rates were also modelled using a Cox proportional hazard model, using the same approach of stepwise selection of the described covariates.

Interactions between the exposure of interest and the other covariates were investigated using interaction terms and tested using likelihood ratio tests. The proportional hazards assumption was assessed using log-log plots for survival.

An explorative analysis of LOS was performed using several accelerated failure time (AFT) survival models, to account for the parametric distribution of the data, with exponential, Weibull, lognormal, and loglogistic distributions considered. All described covariates were considered in the model building process. The fit of these models was subsequently compared using the Akaike information criterion (AIC). The model with the lowest AIC was chosen as the final model for LOS. Results were presented as adjusted time ratios (aTR).

Measures of dispersion were presented as 95% CI where applicable. A *P*-value <0.05 was considered statistically significant. All data were analysed in STATA® v17 (StataCorp, College Station, TX, USA).

## Results

**Fig 1:**
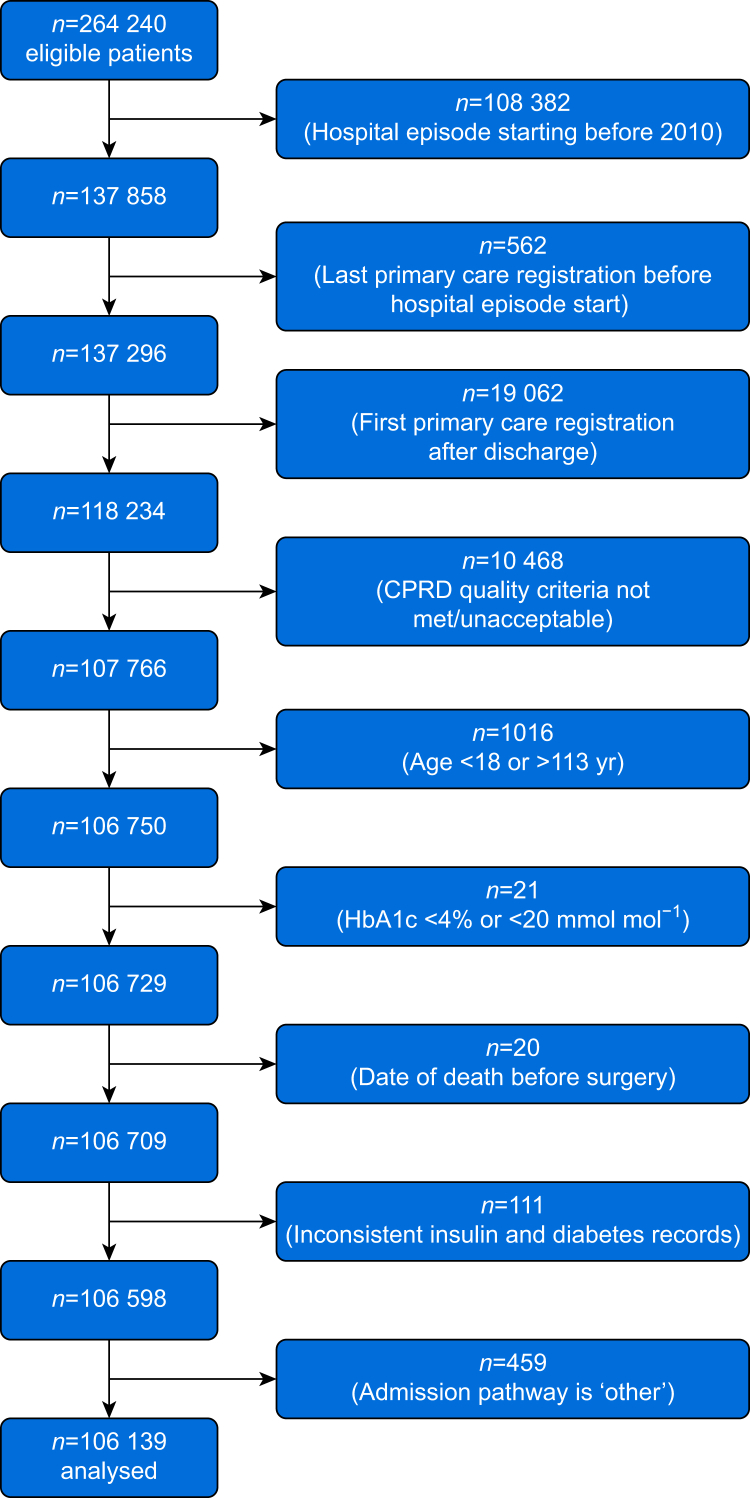
STROBE flow diagram of patient selection. CPRD, Clinical Practice Research Datalink; HbA1c, haemoglobin A1c; STROBE, Strengthening the Reporting of Observational Studies in Epidemiology.

Of the 264 240 eligible patients, a total of 106 139 (49.8% male) with a median (IQR) age of 66 (52–76) yr met the inclusion criteria for final analysis (Fig. 1). The prevalence of diabetes was 10.3% (*n*=10 931), including 653 (6.0%) people with type 1 and 10,278 (94.0%) with type 2 diabetes mellitus. Of all people with diabetes mellitus, 2145 (19.6%) had a record of use of insulin, encompassing all those with type 1 diabetes and 1492 (14.5%) with type 2 diabetes mellitus (Table 1).

**Table 1:** Patient characteristics. IQR, inter-quartile range.

	Without diabetes	With diabetes	*P*-value	Total
Total *n* (%)	95 208 (89.7)	10 931 (10.3)	—	106 931
**Sex**				
Female *n* (%)	48 865 (51.3)	4399 (40.2)	<0.001	53 264 (50.2)
Male n (%)	46 343 (48.7)	6532 (59.8)		52 875 (49.8)
**Age (yr)**				
Median (IQR)	64.7 (50.1–74.9)	72.8 (65.0–79.2)	<0.001	65.8 (51.8–75.6)
**Index of multiple deprivation quintile**				
1 *n* (%)	21 444 (22.5)	2156 (19.7)	<0.001	23 600 (22.2)
2 *n* (%)	20 335 (21.4)	2146 (19.6)		22 481 (21.2)
3 *n* (%)	19 264 (20.2)	2214 (20.3)		21 478 (20.2)
4 *n* (%)	18 034 (18.9)	2244 (20.5)		20 278 (19.1)
5 *n* (%)	16 004 (16.8)	2166 (19.8)		18 170 (17.1)
Missing *n* (%)	127 (0.13)	5 (0.1)		132 (0.12)
**Charlson Comorbidity Index**				
0 *n* (%)	26 179 (27.5)	1071 (9.8)	<0.001	27 250 (25.7)
1 *n* (%)	7947 (8.4)	754 (6.9)		8701 (8.2)
2 *n* (%)	61 082 (64.2)	9106 (83.3)		70 188 (66.1)
**Ethnicity**				
White *n* (%)	87 262 (91.7)	9640 (88.2)	<0.001	96 902 (91.3)
Black *n* (%)	1900 (2.0)	379 (3.5)		2279 (2.2)
Asian *n* (%)	2505 (2.6)	560 (5.1)		3065 (2.9)
Mixed *n* (%)	469 (0.5)	60 (0.6)		529 (0.5)
Unknown *n* (%)	3072 (3.2)	292 (2.7)		3364 (3.2)
**Type of diabetes mellitus**				
Type 1	—	653 (6.0)	—	—
Type 2	—	10 278 (94.0)	—	—
Type 2—no insulin use	—	8786 (85.5)	—	—
Type 2—insulin use	—	1492 (14.5)	—	—
**Urgency of surgery**				
Elective	64 589 (67.8)	7888 (72.2)	<0.001	72 477 (68.3)
Emergency	30 619 (32.2)	3043 (27.8)		33 662 (31.7)
**Operative indication**				
Benign *n* (%)	44 191 (46.4)	3432 (31.4)	<0.001	47 623 (44.9)
Malignant *n* (%)	51 017 (53.6)	7499 (68.6)		58 516 (55.1)
**Surgical access**				
Open *n* (%)	57 834 (60.7)	6943 (63.5)	<0.001	64 777 (61.0)
Minimally invasive *n* (%)	37 374 (39.3)	3988 (36.5)		41 362 (39.0)

### Diabetes mellitus status

#### Ninety-day mortality

**Fig 2:**
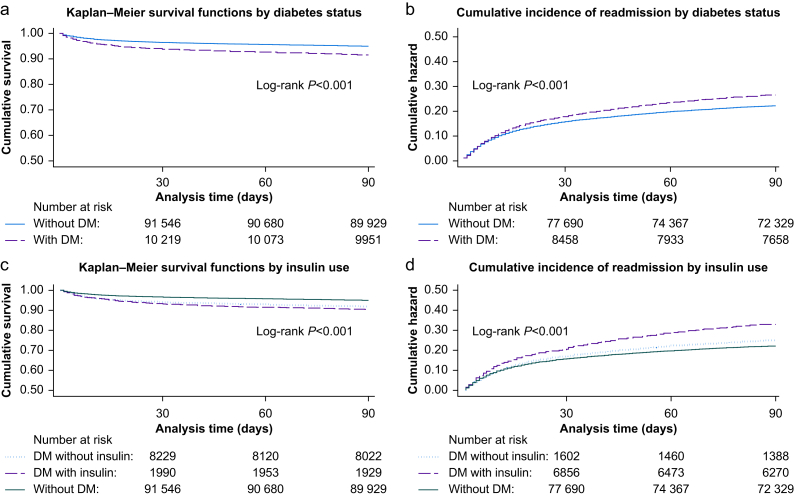
(a) Kaplan–Meier survival functions, stratified by diabetes status. (b) Cumulative incidence of readmission, stratified by diabetes status. (c) Kaplan–Meier survival functions, stratified by use of insulin. (d) Cumulative incidence of readmission, stratified by use of insulin. DM, diabetes mellitus.

The overall unadjusted 90-day mortality risk was 5.7% for the entire cohort, with a decrease in crude mortality rate over time, starting at 7.8% in 2010 and ending at 3.0% in 2020. The overall unadjusted 90-day mortality rate was 226.4 per 1000 person-years (95% CI 220.6–232.4 per 1000 person-years). People with diabetes had a higher 90-day mortality rate (367.8 per 1000 person-years, 95% CI 344.9–392.3 per 1000 person-years) than those without diabetes (212.3 per 1000 person-years, 95% CI 206.3–218.4 per 1000 person-years) (Table 2). Survival functions differed significantly between people with and without diabetes (Fig. 2, *P*<0.001), but not by type of diabetes (Supplementary Fig. S1, *P*=0.799).

**Table 2:** Effect of diabetes status on 90-day mortality. Cox regression model for 90-day mortality with diabetes as the exposure of interest. Deprivation was included in the final model based on likelihood ratio test results (*P*<0.001). CI, confidence interval; HR, hazard ratio.

Covariate	Person- years at risk	*N* Dead (90 days)	90-Day mortality rate per 1000-person years	95% CI	Unadjusted HR	95% CI	*P*-value	Adjusted HR	95% CI	*P*-value
**Sex**										
Male	1.3E+04	2891	231.36	222.98–239.84	1	—	—	—	—	—
Female	1.3E+04	2834	224.52	216.40–232.94	0.98	0.93–1.03	0.491	—	—	—
**Age (yr)**										
16–50	6.2E+03	370	59.35	53.60–65.71	1	—	—	1	—	—
51–60	4.0E+03	539	133.94	123.09–145.73	2.25	1.97–2.58	<0.001	2.36	2.06–2.70	<0.001
61–70	5.9E+03	1182	200.70	189.57–212.47	3.36	2.99–3.78	<0.001	3.66	3.25–4.14	<0.001
71–80	6.0E+03	1825	304.08	290.45–318.36	5.08	4.54–5.69	<0.001	5.25	4.67–5.90	<0.001
>80	3.0E+03	1809	608.33	580.93–637.02	9.99	8.93–11.12	<0.001	8.82	7.84–9.93	<0.001
**Index of multiple deprivation quintile**										
1	5.6E+03	1186	211.36	199.66–223.73	1	—	—	1	—	—
2	5.3E+03	1100	206.21	194.38–218.77	0.97	0.89–1.06	0.446	0.99	0.91–1.07	0.764
3	5.1E+03	1170	230.25	217.43–243.83	1.09	1.00–1.18	0.038	1.08	1.00–1.17	0.064
4	4.8E+03	1149	240.03	226.55–254.32	1.14	1.05–1.23	0.002	1.18	1.09–1.28	<0.001
5	4.3E+03	1108	258.95	244.14–274.66	1.23	1.13–1.33	<0.001	1.23	1.14–1.34	<0.001
**Charlson Comorbidity Index**										
0	6.6E+03	938	143.03	134.16–152.48	1	—	—	1	—	—
1	2.1E+03	382	183.84	166.30–203.23	1.28	1.13–1.44	<0.001	1.17	1.04–1.32	0.011
2	1.6E+04	4405	267.17	259.39–275.17	1.84	1.72–1.98	<0.001	1.79	1.66–1.93	<0.001
**Diabetes mellitus**										
No	2.3E+04	4798	212.27	206.34–218.36	1	—	—	1	—	—
Yes	2.5E+03	927	367.84	344.91–392.30	1.72	1.60–1.84	<0.001	1.28	1.19–1.37	<0.001
**Urgency of surgery**										
Elective	1.8E+04	1519	86.43	82.19–90.89	1	—	<0.001	1	—	—
Emergency	7.4E+03	4147	556.88	540.19–574.09	6.25	5.89–6.63	<0.001	4.83	4.52–5.17	<0.001
**Operative indication**										
Benign	1.1E+04	3235	292.95	283.03–303.22	1	—	—	1	—	—
Malignant	1.4E+04	2438	174.13	167.36–181.18	0.60	0.57–0.63	<0.001	0.77	0.73–0.82	<0.001
**Surgical access**										
Open	1.5E+04	5149	342.48	333.25–351.96	1	—	—	1	—	—
Minimally invasive	1.0E+04	576	57.09	52.61–61.95	0.17	0.16–0.19	<0.001	0.4	0.35–0.42	<0.001

The results of the univariable and multivariable regression analysis of 90-day mortality, with diabetes status as the exposure of interest, are presented in Table 2. The risk of 90-day mortality was significantly increased in people with diabetes (aHR 1.28, 95% CI 1.19–1.37, *P*<0.001) compared with those without diabetes. There were no significant interactions between the exposure of interest and each covariate, including comorbidity burden. The proportional hazards assumption was met for the exposure of interest (Supplementary Fig. S2). The increased risk of 90-day mortality for people with diabetes was similar across those undergoing either emergency (aHR 1.28, 95% CI 1.18–1.40, *P*<0.001) or elective colorectal surgery (aHR 1.23, 95% CI 1.07–1.40, *P*<0.001) (Supplementary Table S3).

#### Ninety-day readmission

Emergency readmission within 90 days of discharge occurred in 20 542 (19.4%) of all patients and was more common in people with diabetes compared with those without diabetes (21.7% *vs* 19.1%, *P*<0.001). People with type 1 diabetes experienced 90-day readmission more frequently than those with type 2 diabetes (26.3% *vs* 21.4%, *P*=0.003).

The overall 90-day readmission rate was 974.2 per 1000 person-years (95% CI 960.9–987.6 per 1000 person-years). The rate of 90-day readmission was more common in people with diabetes (1152.1 per 1000 person-years, 95% CI 1106.6–1199.5 per 1000 person-years) compared with those without diabetes (954.9 per 1000 person-years, 95% CI 941.1–968.9 per 1000 person-years) (Fig. 2).

Results from the regression analysis of 90-day readmission, with diabetes as the exposure of interest, are presented in Table 3. People with diabetes demonstrated increased 90-day readmission rates in both the unadjusted (HR 1.19, 95% CI 1.14–1.24, *P*<0.001) and adjusted (aHR 1.23, 95% CI 1.18–1.29, *P*<0.001) Cox regression models. Log-log plots for readmission by diabetes status showed crossover between the groups of people with and without diabetes (Supplementary Fig. S3).

**Table 3:** Effect of diabetes status on 90-day readmission. Cox regression model for 90-day readmission with diabetes as exposure of interest. Deprivation was included in the final model based on likelihood ratio test results (*P*<0.001). CI, confidence interval; HR, hazard ratio.

Covariate	Unadjusted HR	95% CI	*P*-value	Adjusted HR	95% CI	*P*-value
**Sex**						
Male	1	—	—	1	—	—
Female	0.96	0.94–0.99	0.009	0.95	0.92–0.98	<0.001
**Age (yr)**						
16–50	1	—	—	1	—	—
51–60	0.92	0.88–0.96	<0.001	0.92	0.88–0.97	<0.001
61–70	0.82	0.79–0.86	<0.001	0.83	0.80–0.87	<0.001
71–80	0.83	0.80–0.86	<0.001	0.83	0.79–0.86	<0.001
>80	0.84	0.80–0.88	<0.001	0.83	0.78–0.87	<0.001
**Index of multiple deprivation quintile**						
1	1	—	—	1	—	—
2	1.04	1.00–1.08	0.08	1.03	0.98–1.07	0.214
3	1.05	1.01–1.10	0.027	1.02	0.98–1.06	0.3999
4	1.15	1.10–1.20	<0.001	1.10	1.05–1.15	<0.001
5	1.28	1.23–1.34	<0.001	1.19	1.14–1.25	<0.001
**Charlson Comorbidity Index**						
0	1	—	—	1	—	—
1	0.98	0.92–1.03	0.419	1.07	1.06–1.19	0.049
2	1.05	1.02–1.08	0.003	1.23	1.23–1.32	<0.001
**Diabetes mellitus**						
No	1	—	—	1	—	—
Yes	1.19	1.14–1.24	<0.001	1.23	1.18–1.29	<0.001
**Urgency of surgery**						
Elective	1	—	—	1	—	—
Emergency	1.39	1.35–1.43	<0.001	1.27	1.23 to 1.31	<0.001
**Operative indication**						
Benign	1	—	—	1	—	—
Malignant	0.74	0.71–0.76	<0.001	0.84	0.78–0.86	<0.001
**Surgical access**						
Open	1	—	—	1	—	—
Minimally invasive	0.77	0.75–0.80	<0.001	0.87	0.84–0.89	<0.001

#### Length of stay

The median (IQR) hospital LOS for the entire cohort and for people without diabetes was 8 (5–15) days. In those with diabetes, the median (IQR) LOS was 9 (6–17) days. A log-logistic AFT model was used to evaluate LOS with the best goodness of fit compared with other distributions (Supplementary Table S4). All covariates listed previously were considered in this model and were each shown to be statistically significant. After adjusting for these covariates, people with diabetes experienced a 10% longer length of hospital stay compared with those without diabetes (aTR 1.10, 95% CI 1.08–1.11, *P*<0.001) (Table 4).

**Table 4:** Effect of diabetes status on length of stay. CI, confidence interval.

Covariate	Adjusted time ratio	Standard error	95% CI	*P*-value
**Sex**				
Male	1	—	—	—
Female	0.96	0.00	0.95–0.97	<0.001
**Age (yr)**				
16–50	1	—	—	—
51–60	1.04	0.01	1.03–1.06	<0.001
61–70	1.09	0.01	1.08–1.10	<0.001
71–80	1.21	0.01	1.20–1.23	<0.001
>80	1.44	0.01	1.41–1.46	<0.001
**Index of multiple deprivation quintile**				
1	1	—	—	—
2	1.01	0.01	1.00–1.02	0.029
3	1.05	0.01	1.03–1.06	<0.001
4	1.08	0.01	1.07–1.10	<0.001
5	1.13	0.01	1.12–1.15	<0.001
**Charlson Comorbidity Index**				
0	1	—	—	—
1	1.05	0.01	1.03–1.06	<0.001
2	1.13	0.01	1.12–1.15	<0.001
**Diabetes mellitus**				
No	1	—	—	—
Yes	1.10	0.01	1.08–1.11	<0.001
**Urgency of surgery**				
Elective	1	—	—	—
Emergency	1.88	0.01	1.86–1.90	<0.001
**Operative indication**				
Benign	1	—	—	—
Malignant	0.84	0.00	0.83–0.85	<0.001
**Surgical access**				
Open	1	—	—	—
Minimally invasive	0.7	0.00	0.70–0.71	<0.001

### Use of insulin

#### Ninety-day mortality

Use of insulin in people with diabetes mellitus was associated with the highest 90-day mortality rates (423.8 per 1000 person-years, 95% CI 370.0–485.3 per 1000 person-years), compared with both those with diabetes without use of insulin (354.2 per 1000 person-years, 95% CI 329.3–381.1 per 1000 person-years) and those without diabetes (212.3 per 1000 person-years, 95% CI 206.3–218.4 per 1000 person-years) (Supplementary Table S5, Fig. 2).

The results of the regression analysis of 90-day mortality, with use of insulin considered the primary exposure of interest, are presented in Supplementary Table S5. Use of insulin in people with diabetes was significantly associated with increased 90-day mortality risk (aHR 1.51, 95% CI 1.31–1.74, *P*<0.001) over and above the increased risk experienced by those with diabetes (aHR 1.22, 95% CI 1.13–1.33, *P*<0.001), with reference to those without diabetes as a baseline group. Log-log plots, stratified by use of insulin, showed crossover between the group of people with diabetes with use of insulin and those with diabetes without use of insulin (Supplementary Fig. S4).

#### Ninety-day readmission

A higher proportion of people with diabetes mellitus and use of insulin was readmitted within 90 days of discharge, compared with those with diabetes without use of insulin (26.0% *vs* 19.2%, *P*<0.001). Use of insulin in people with diabetes was associated with higher 90-day readmission rates (1453.9 per 1000 person-years, 95% CI 1338.0–1579.0 per 1000 person-years), over and above those with diabetes without use of insulin (1082.9 per 1000 person-years, 95% CI 1034.1–1134.0 per 1000 person-years) and those without diabetes (954.9 per 1000 person-years, 95% CI 941.1–968.9 per 1000 person-years) (Fig. 2).

People with diabetes and use of insulin were 1.5 times more likely to be readmitted within 90 days (aHR 1.46, 95% CI 1.34–1.59, *P*<0.001), whereas those with diabetes without use of insulin were 1.2 times more likely to be readmitted within 90-days (aHR 1.17, 95% CI 1.12–1.23, *P*<0.001), compared with those without diabetes (Supplementary Table S6). Log-log plots for readmission by use of insulin are presented in Supplementary Figure S5.

## Discussion

In this analysis of patients undergoing colorectal resections, those with diabetes mellitus experienced a significantly increased risk of 90-day mortality, prolonged LOS, and 90-day readmission, compared with those without diabetes mellitus. People with diabetes and use of insulin appeared to incur an increased risk of poor postoperative outcomes compared with those with diabetes without use of insulin and those without diabetes. These results are in concordance with findings in other medical and surgical specialties, whilst representing new evidence in the field of colorectal surgery.^23^, ^24^, ^25^, ^26^

### Current literature

The overall 5.7% 90-day mortality rate described in this cohort lies within the range described in current literature (4.4–11%).^27^, ^28^, ^29^, ^30^, ^31^ Although this outcome is limited to short-term postoperative events, it has been shown to correlate with longer-term mortality, whilst still reflecting outcomes related to the surgical intervention.^31^

Birch and colleagues^6^ investigated postoperative outcomes for people with diabetes undergoing surgery for colorectal malignancy only and found an increased unadjusted 90-day mortality risk limited to people with diabetes and resulting complications (6.6% *vs* 3.2% in those without diabetes), but not for those with uncomplicated diabetes. This was reiterated in their adjusted analysis of 1-yr postoperative mortality (OR 1.05, 95% CI 0.99–1.11 for people with uncomplicated diabetes, OR 1.58, 95% CI 1.51–1.66 for those with complicated diabetes). Of note, almost half (49.2%) of their population with diabetes was classed as having complicated diabetes, which may have contributed to 1-yr mortality independent of the surgical intervention. This cohort also represents a period of time (2005–16) which only partially includes diabetes-specific perioperative guidelines.^9^^,^^15^ These may have resulted in improved perioperative optimisation and care, and patient selection, leading to a more homogenous group of people with diabetes undergoing colorectal surgery. Nevertheless, consideration of diabetes-related complications may be important in the ongoing care for people with diabetes undergoing surgery for colorectal malignancy.

A recent meta-analysis by Tan and colleagues^5^ reported no statistically significant difference in 30-day mortality (OR 2.65, 95% CI 0.80–8.70, *P*=0.109) after colorectal surgery for people with diabetes compared with those without diabetes, based on a pooled analysis of three cohort studies (*n*=3526 [11.3%] with diabetes). However, these results may not represent the true risk, as this study appears to be underpowered to detect a difference in 30-day mortality (a more infrequent outcome), based on a relatively small sample size and indicated by the wide CI surrounding the effect size.

Tan and colleagues^5^ also performed a pooled meta-analysis of hospital readmission rates after colorectal surgery, derived from two studies (*n*=181 876 total patients, *n*=24 396 [13.4%] with diabetes), and reported an increased risk of readmission after colorectal surgery for people with diabetes (OR 1.41, 95% CI 1.35–1.47, *P*<0.001). Although the evidence in patients undergoing colorectal surgery is limited, an increased readmission risk for people with diabetes has been consistently described in other surgical^24^^,^^25^^,^^32^ and medical^33^, ^34^, ^35^ specialities. An increased 30- and 90-day readmission risk has also been demonstrated for people with diabetes and use of insulin over and above those with diabetes without use of insulin.^24^^,^^33^

Lastly, LOS has been shown to be increased for people with diabetes in other noncardiac surgical specialties.^24^^,^^26^ The reason for this is likely multifactorial, with possible contributing factors including direct issues with blood glucose control after surgery, or diabetes-related complications such as surgical site infections and poor wound healing.^5^ Within colorectal surgery, Cologne and colleagues^36^ report a trend towards increased hospital stay in people with diabetes hospitalised with diverticulitis (mean 7.29 *vs* 5.81 days, *P*=0.05), although their study was not limited to surgical patients and did not report an adjusted effect size. It has also been shown that general surgical patients with diabetes and use of insulin are prone to increased LOS (excess LOS 1.2 days) compared with those with diabetes and without use of insulin (excess LOS 0.9 days) and those without diabetes.^26^

### Clinical significance

Diabetes mellitus increased the risk of 90-day mortality by 28% (aHR 1.28, 95% CI 1.19–1.37) and 90-day readmission by 23% (aHR 1.23, 95% CI 1.18–1.29) in patients undergoing colorectal resections, whilst also increasing LOS by 10% (aTR 1.10, 95% CI 1.08–1.11), compared with people without diabetes mellitus. Use of insulin in people with diabetes was associated with an additional risk of worse outcomes across all three measures. These findings should be considered during shared decision-making discussions and when assessing the suitability of each person for surgery. Both increased readmission rates and prolonged hospital stay translate not only to delayed patient recovery and increased morbidity, but also to decreased bedspace availability in hospitals and increased healthcare expenditure. This, in turn, will impact on preoperative planning of service provision and resource allocation for patients undergoing colorectal surgery. Adherence to current perioperative guidelines and patient education is essential to mitigate the risk of poor outcomes in this vulnerable group of patients.^9^^,^^12^^,^^13^

### Strengths and limitations

This study represents a national cohort encompassing a substantial number of patients, followed over a 10-yr period, with linked data captured through national primary and secondary care databases. To our knowledge, this study describes the largest cohort of people with diabetes mellitus undergoing colorectal surgery, and associated use of insulin, and subsequent postoperative outcomes, to date.

Although several measures have been taken to reduce bias, this observational study depends largely on accurate coding within the adopted registries to identify the study population, exposure variables, and outcomes, which are subject to human error at source. Furthermore, this study encompasses a long period of time, throughout which there have been developments in perioperative care, surgical approach, and guidelines relating to the management of people with diabetes undergoing surgery.^9^^,^^12^, ^13^, ^14^, ^15^ These changes may have selectively affected patients undergoing colorectal surgery and subsequent postoperative outcomes. This is reflected by the decrease in crude mortality rate over time observed both in this cohort and current literature. Data pertaining to perioperative factors such as inpatient prescribing during hospital stay or Enhanced Recovery After Surgery (ERAS) implementation and compliance are not recorded in HES and were, therefore, not included in our analysis.

Additional potential confounders that were not available for analysis include tumour staging and peritoneal soiling. Furthermore, group sizes and event numbers per group were insufficient to perform subgroup analysis of insulin use stratified by type of diabetes, and differentiate people with type 1 diabetes from those with type 2 diabetes and insulin use. Although the survival curves stratified by type of diabetes did not show a significant difference, there may still be a difference in postoperative outcomes between these groups, which warrants further investigation when larger numbers are available.

### Future research

In this study, insulin use was used as a marker of disease severity for diabetes mellitus. Further investigation is warranted into whether other measures of disease severity and glycaemic control, such as diabetes-related complications, haemoglobin A1c (HbA1c) concentrations, and perioperative hyperglycaemia, are able to additionally stratify the mortality risk of people with diabetes undergoing colorectal surgery, to help guide clinical decision-making in daily practice. Additionally, there would be value in exploring the longer-term impact of diabetes on postoperative outcomes in patients undergoing colorectal resections. Lastly, local or regional audit of compliance with perioperative guidelines regarding the management of people with diabetes mellitus undergoing colorectal surgery is encouraged.

## Conclusions

People with diabetes mellitus undergoing colorectal surgery are at a higher risk of 90-day mortality, prolonged length of stay, and 90-day readmissions, compared with those without diabetes. Use of insulin in people with diabetes mellitus is associated with an additional risk of poor postoperative outcomes. Awareness of these increased risks should be considered during the perioperative optimisation and care of the patient, and the patient selection process.

## Author contributions

Study design: SG, CL-L, DNL, CJC, DJH

Data analysis: SG, CJC, DJH

Data interpretation: SG, CL-L, DNL, CJC, DJH

Writing of the manuscript: SG, CL-L, DNL, CJC, DJH

Critical review: DNL, CJC, DJH

Final approval: SG, CL-L, DNL, CJC, DJH

Agreement to be accountable for all aspects of the work thereby ensuring that questions related to the accuracy or integrity of any part of the work are appropriately investigated and resolved: SG, CL-L, DNL, CJC, DJH

## Declaration of interest

DNL has received an unrestricted educational grant from B. Braun for unrelated work. He has also received speaker's honoraria for unrelated work from Abbott, Nestlé and Corza. All other authors declare that they have no conflicts of interest.

## Funding

The Medical Research Council (MR/K00414X/1), Arthritis Research UK (19891), and the National Institute for Health Research Nottingham Biomedical Research Centre (NIHR203310). The funders had no role in the design or conduct of the work, or in the decision to publish. This paper presents independent research. The views expressed are those of the authors and not necessarily those of the funders, NHS, or the Department of Health.

## Data sharing statement

Access to the Clinical Practice Research Datalink and linked data was provided under a licence to the University of Nottingham. Under the terms and agreement of this licence, data cannot be provided for the purposes of sharing.

## Generative artificial intelligence

None used.
