# Supplementary material for: The effect of diabetes mellitus on perioperative outcomes after colorectal resection: a national cohort study
Source: Br J Anaesth. 2024 May 16;133(1):67–76. doi: 10.1016/j.bja.2024.04.010 (PMC11213983; doi:10.1016/j.bja.2024.04.010)
Supplement: Multimedia component 1 [file mmc1.docx]

**SUPPLEMENTAL DATA**

**Table of contents**

Supplementary Table S1: Code Lists page 2-4

Supplementary Table S2: Covariates page 5

Supplementary Table S3: Effect of diabetes on 90-day mortality by surgical urgency page 6-7

Supplementary Table S4: Comparison of Accelerated Failure Time (AFT) models page 8

Supplementary Table S5: Effect of insulin use on 90-day mortality page 9-10

Supplementary Table S6: Effect of Insulin use on 90-day readmission page 11

Supplementary Figure S1: Kaplan Meier survival functions stratified by type of diabetes page 12

Supplementary Figure S2: Log-log plots for survival, stratified by diabetes status page 13

Supplementary Figure S3: Log-log plots for readmission, stratified by diabetes status page 14

Supplementary Figure S4: Log-log plots for survival, stratified by use of insulin page 15

Supplementary Figure S5: Log-log plots for readmission, stratified by use of insulin page 16

**Supplementary Table S1: Code lists**

| Codes used to identify colectomy, inflammatory bowel disease (IBD), diverticular disease (DD), and minimally-invasive surgery (MIS)  **Colectomy (**OPCS-4)  H041, H042, H043, H048, H049, H051, H052, H053, H058, H059, H061, H062, H063, H064, H068, H069, H071, H072, H073, H074, H078, H079, H081, H082, H083, H084, H085, H088, H089, H091, H092, H093, H094, H095, H098, H099, H101, H102, H103, H104, H105, H108, H109, H111, H112, H113, H114, H115, H118, H119, H291, H292, H293, H294, H298, H299, H331, H332, H333,  H334, H335, H336, H337, H338, H339  **IBD (**ICD-10 codes)  K50, K500, K501, K508, K509, K51, K510, K512, K513, K514, K515, K518, K519, K520, K521, K522, K523, K528, K529  **DD (**ICD-10 codes)  K57, K570, K571, K572, K573, K574, K575, K578, K579  **MIS** (OPCS-4 codes)  Y50.8, Y57.1, Y75.2, Y75.3 |
| --- |
| Codes used to identify diabetes mellitus (DM) and Insulin-use  **DM** from HES data (ICD-10)  E100, E101, E102, E103, E104, E105, E106, E107, E108, E109, E110, E111, E112, E113, E114, E115, E116, E117, E118, E119, E120, E121, E122, E123, E124, E125, E126, E127, E128, E129, E130, E131, E132, E133, E134, E135, E136, E137, E138, E139, E140, E141, E142, E143, E144, E145, E146, E147, E148, E149, O240, O241, O242, O243, O244, O249  **DM** from CPRD data (Original Read Codes)  66AJ1, C10E, F4201, 66A8, C10F, 1434, 8H3O, C10F4-1, C10A1, C10E5-1, C10E9, 9OL2, C10E9-1, C10F3, C10A7, 9OL1, F3721, C10F3-1, F3720, F3722, 66AD, C10E5, C10F4, K01x1, 66A9, 66AT, F4203, L1806, L1807, M2711, M0372, M2710, M2712, K08yA-1, 9N2i, K08yA, C10F9, C10EA-1, C10EE-1, C10EH-1, C10FF, C10E4-1, C10E4, C10EA, C10FG, C10EH, C10D-1, 2G5A, 3881, C10FH-1, C10FH, 2BBV, F3813, 9N1v, C109G, 2BBT, F4206, 2G5B, 14F4, 9OLB, C109J-1, 2G5E, 2BBQ, 7276, 8CP2, 2G5G, F4207, 2G5J, 8Hj0, 2G5W, 2BBS, C10FK, C10F-1, C10E-1, C10EL, 2G5H, 2G5L, 9NN8, 8HHy, 9M00, 8HTi, 9N4p, 9N0o, 2G5F, 9N1i, 9360, C10M0, 9N0n, 9NND, 8Hj4, 9OLJ, 9OLG, 8I82, 8I81, 8Hj3, 9NiA, 9NiC, C10FS, 9OLL, 2BBr, 9m06, 9m08, 9N0m, EMISR4QIN2, L180-99, EMISQLE12, EMISQLE17, EMISQLE9, EMISQRI13, EMISQRI14, EMISQLA1, EMISQLE11, EMISQRI15, EMISQLE13, EMISQRI8, EMISQLE15, EMISQLE8, EMISQRI16, EMISQRI17, EMISQRI11, EMISQLE10, EMISQLE16, EMISQRI12, ALLERGY3148, C10A-1, EMISNQIN24, 66AQ1, 66At1-1, 66At0, 66At1, 2G5e, EMISNQRE56, EMISNQIN23, EMISNQIN25, EMISNQIN27, EMISNQIN33, 9NiZ, EMISNQSE63, EMISNQDI79, EMISNQIN165, EMISNQIN162, EMISNQDA12, EMISNQAD61, EMISNQHO54, EMISNQAC841, EMISNQIN163, EMISNQIN164, EMISNQUN46, EMISNQPA381, 8HTE1, 8OA3, K27y7, 9NJy, 8Hlc, 679L2, 9NJy0, 66o3, EMISNQ3D9, 66o1, EMISNQLI60, 9NJy1, 66AH2, ESCTSE11, ESCTMA7, ESCTDI18-1, ESCTPH1, ESCTIN27, ESCTME15, ESCTLI13, 9NlP1, EMISNQNE23, ESCTAB10, ESCTIN27-1, 67HA, 9NN91, 67D8, EMISNQLI56, EMISNQLI58, ESCTHY3, EMISNQLI59, 9NN90, ESCTHY2, C1001-2, EMISNQHO137, C10F7-1, C10E8-1, EMISNQKE155, Lyu29, R0542, EMISNQLI57, Kyu03, C104y, C10C-2, R0543, C10EJ-1, C10E8, Cyu20, C108-2, C108E-2, C1083-2, C108D-2, C1080-2, C1087-2, C1089-2, C109-2, C1099-2, C1082-1, C1080-1, C109G-1, C108y, C109C-2, C1091-2, C108D-1, C1090-1, 9h42, C109B, C109, C10FR, C10zy, C11y0, 679L, C1088-2, C109B-2, C1083-1, C108A-1, C109-3, C1094-1, C108H-2, C108C-2, C108A-2, C1097-2, C1096-2, C108E-1, C108C-1, C1085-1, C1096-1, C1097, C108J-2, C109G-2, C109F-2, C1088-1, C108H-1, C109E-1, 8CA41, Q440, C10G0, C109D-2, C1090-2, C1094, C109E, L180X, C1094-2, C108B-1, C109F-1, C109A, C1085-2, C1092-2, C108F-1, C1093-1, C109C-1, C10FD, TJ23, Cyu22, C1096, 2BBF, C10N0, C1090, 66AA-1, C10EQ-1, 9m07, PKyP, 8I84, 9m0D, C1091, 67W1, C10Q, L1805, C10EA-2, C108E, C10ED-2, C10EH-2, C108F, C108J, C108-1, C10E3-2, C108B, C108C, C108G, C10E9-2, C10EB-2, C10EJ-2, C1087, C10E7-2, C10FR-1, 8I83, 9NiD, 8IEa, 679L2-1, C108z, C108A, C1089, C10ED-1, C10FM, C10EP, C10E7, C10EF-1, C10FD-1, C10F0-1, C10F2, C10FA-1, C10FB-1, ^ESCTNO698127, C10M, C10EE-2, 2G510, C108H, C10EG, C1084, C1081-1, C108G-1, 9OLP, Cyu23, C10EG-2, 2G5C, C1082, C1080, C108, C10E1-2, C10EC-2, C10EF-2, C10E6-1, ^ESCTHY812040, 8H2J, ^ESCTLU786380, C10F0, C10FC, C10FC-1, C10EK-1, C10EL-1, C10FL, C1083, C1000-1, C10E0-2, 2BBW, 679R, ^ESCTLU786381, ^ESCTPR351158, ^ESCTDE781181, C109J-2, C10E3-1, C10EK, C10FN, 2BBl, ZV653-2, C10F5-1, F4208, C109K, C10ED, C10F6-1, C10FE, C10EF, C10FQ, C10E1, C1095-2, C109J, 66AX, C10E3, C10E7-1, C10EC, C10F1, C10F2-1, C10FN-1, C10EP-1, C1095-1, C1085, ^ESCTRA786157, ^ESCT1169219, ^ESCTDI796695, C10E6, C10AW, ^ESCTAT1163321, ^ESCT1169220, ^ESCTPR803330, C10A4, C10A5, C10EB, C10FA, C1086-2, ^ESCTGI764468, C10A2, ^ESCTAD1163322, ^ESCTAN796694, 66Af, C106y, 2BBX, C109D, C10EQ, C109H-1, C10A, L180B, C10FP-1, EMISNQLI61, ^ESCTPR750021, C10A3, C10AX, C10FG-1, F1711, EMISNQNE127, ESCTPR17, C10F6, C10EM-1, C10EN-1, C10FL-1, C10FM-1, C10FP, C10FQ-1, C109B-1, C1081, C10E8-2, C10EG-1, 8OAL, 8Hj5, 66AN, C1084-1, C109E-2, C10FF-1, F4200, 2BBP, C10E2, C10F7, C1087-1, 9m0C, C10E-2, C10E2-2, C108B-2, C1092, C108J-1, C103y, C108-3, C10EC-1, C10EN, C105y, EMISNQIN28, C1086, C10E6-2, C10F1-1, C10F5, C1089-1, C109D-1, 66At0-1, 2G5d, 8BL2, 93C4, 9NN9, 2G5I, EMISNQRE335, C10yz-97, 2BBL, 9M10, C10FJ-1, F372, C108D, C10N, 9OLK, EMISTO1, EMISNQSI2, C10E5-2, ^ESCTPR803329, ^ESCTAN796693, C10EJ, C10FJ, ^ESCTGI774616, C108F-2, C1095, ESCTPR17-1, EMISNQIN34, C109H-2, C109A-1, C10N1, L180A, C109A-2, C10C-1, C107y, Cyu21, C1091-1, C1099-1, 67IJ1, 2BBk, C10yy, C10E4-2, C1082-2, C1086-1, C10A6, EMISQRI9, 9N1o, C10FK-1, 9OLH, U6023-E, 9m0E, C10E0-1, C10E2-1, C1081-2, C10E1-1, TJ23z, C1093-2, C1097-1, C10E0, C10FB, C10A0, F4205, EMISNQIN35, EMISNQDE12, F4202, 8H7r, C10F9-1, C10G, 2BBR, 9m0B, EMISNQIN30, C10EB-1, C10FE-1, C10EM, 9m0A, EMISQRI10, 9m05, C10EE, 2BBo, 2G5V, 2BBG, TJ23-99, C1099, C101y, C1093, U6023-1, C108G-2, C1088, 679L0, EMISQLE14, 66AJ-1, C1084-2, EMISNQCO167, 8HTk, 2G5K, 9N2d, 8Hl1, 9m00, C109-1  **Insulin** (Dictionary of Medicines and Devices Codes)  3284211000001102, 10250211000001100, 400780006, 30172211000001104, 11933011000001106, 3283211000001100, 400877001, 30171811000001104, 36082811000001104, 3468611000001108, 3279211000001105, 34043211000001100, 3281611000001102, 3282211000001106, 3468711000001104, 19570211000001104, 34043011000001104, 3277811000001103, 3277211000001104, 3268711000001103, 3264711000001106, 3269711000001107, 3273911000001104, 3270211000001103, 3271711000001102, 3267811000001107, 3264411000001100, 3263611000001100, 3266111000001103, 3264111000001105, 3259811000001109, 18150311000001108, 3263011000001107, 3275311000001104, 3274811000001107, 3275011000001102, 3273111000001102, 3273611000001105, 3271611000001106, 3272211000001102, 3270511000001100, 9437511000001100, 17609511000001108, 3285511000001103, 3281211000001104, 3272011000001107, 3273411000001107, 3271011000001104, 3272411000001103, 3272811000001101, 3278611000001103, 3278311000001108, 3274511000001109, 3277711000001106, 3266811000001105, 3267911000001102, 3280011000001100, 3472311000001106, 3259411000001107, 3255911000001101, 3256111000001105, 3472511000001100, 3258411000001104, 3473011000001104, 3284011000001107, 3333111000001107, 3276011000001106, 13884711000001104, 13884911000001102, 3276911000001105, 3275711000001100, 3280611000001107, 4029411000001100, 4028311000001101, 4033211000001103, 4028811000001105, 4053611000001100, 4034311000001108, 4034911000001109, 7594211000001102, 7589411000001100, 11148111000001100, 7597611000001106, 7589911000001108, 9532111000001100, 9528311000001104, 10898411000001104, 10097211000001102, 10093311000001104, 12144611000001100, 10344911000001108, 10690511000001104, 10690811000001108, 13859411000001100, 18030311000001100, 15603111000001104, 16530311000001108, 18083911000001104, 18046311000001104, 21939511000001100, 21928511000001108, 21930011000001100, 21939611000001100, 21939711000001104, 21931911000001108, 26655811000001104, 26209611000001108, 28279611000001108, 28054311000001104, 28989711000001104, 28926811000001100, 29903611000001104, 29866811000001104, 35214311000001104, 3280711000001103, 36047011000001104, 34043411000001104, 3287911000001100, 36047111000001104, 9528811000001108, 36047211000001104, 3263711000001109, 3264211000001104, 3265011000001108, 3261411000001102, 3262511000001106, 3262011000001103, 3260611000001109, 3260811000001108, 17608511000001104, 19354411000001104, 3282711000001104, 36047511000001104, 3280111000001104, 36047611000001104, 35776411000001104, 3284311000001105, 35776511000001104, 36047711000001104, 3278511000001102, 13877811000001104, 36047811000001104, 35776811000001100, 35216811000001100, 3269911000001109, 36047911000001110, 3271311000001101, 3309511000001105, 3312111000001108, 3312411000001103, 3312611000001100, 3310711000001105, 3311311000001101, 3311611000001106, 3291711000001105, 3288511000001106, 3290411000001108, 3294911000001105, 3285011000001106, 3285611000001104, 36048711000001104, 3282311000001103, 3281811000001103, 3284911000001106, 3284111000001108, 36620611000001104, 36630511000001104, 36630611000001110, 36618311000001110, 36911311000001104, 36931811000001104 |

**Supplementary Table S2: Covariates**

| Sex | Categorised into either male or female. |
| --- | --- |
| Age | Categorised into groups of ages 16 to 50, 51 to 60, 61 to 70, 71 to 80 and more than 80 years old. |
| Deprivation | As defined by the Index of Multiple Deprivation (IMD) 2015, which is a measure of relative deprivation across the UK. The IMD divides deprivation into five quintiles based on relative disadvantage, with quintile 1 being the most deprived and quintile 5 being the least deprived. |
| Comorbidity | Classified according to the Charlson Comorbidity Index, and categorised into 0, 1, and ≥2. |
| Diabetes status | Categorised into patients with DM or without DM. Patients with missing diabetes and insulin records were classified as not having diabetes. |
| Use of insulin | Classed as either patients with DM with Insulin-use, patients with DM without Insulin-use or patients without DM (as a reference group). |
| Urgency of surgery | Categorised as either elective or emergency surgery, according to the admission classification recorded for the surgical procedure. |
| Operative indication | Defined as the underlying reason for colorectal resection, and classed as either benign or malignant. |
| Surgical access | Referring to the surgical approach used to perform the colorectal resection and categorised into either open (laparotomy) or minimally-invasive (laparoscopic and robotic procedures). |
| Missing values were presented as a separate group where applicable but were not considered an individual covariate in regression analysis. | |

**Supplementary Table S3: Effect of diabetes on 90-day mortality by surgical urgency. CI, confidence interval; HR, hazard ratio.**

| **A) Emergency colorectal resections** | | | | | | | |
| --- | --- | --- | --- | --- | --- | --- | --- |
| **Covariate** | **N Dead (90 days)** | **Unadj. HR** | **95% CI** | ***P*-value** | **Adj. HR** | **95% CI** | ***P*-value** |
| **Sex** | | | | | | | |
| Male | 1894 | 1 | – | – | 1 | – | – |
| Female | 2253 | 1.03 | 1.03–1.17 | 0.002 | 0.99 | 0.93–1.05 | 0.799 |
| **Age (years)** |  |  |  |  |  |  |  |
| 16-50 | 295 | 1 | – | – | 1 | – | – |
| 51-60 | 419 | 2.82 | 2.43–3.27 | <0.001 | 2.4 | 2.07–2.79 | <0.001 |
| 61-70 | 834 | 4.47 | 3.92–5.11 | <0.001 | 3.46 | 3.02–3.95 | <0.001 |
| 71-80 | 1254 | 6.36 | 5.61–7.22 | <0.001 | 4.57 | 4.01–5.21 | <0.001 |
| >80 | 1345 | 10.63 | 9.37–12.06 | <0.001 | 7.41 | 6.50–8.44 | <0.001 |
| **Index of multiple deprivation quintile** | | | | | | | |
| 1 | 852 | 1 | – | – | – | – | – |
| 2 | 772 | 0.92 | 0.83–1.01 | 0.081 | – | – | – |
| 3 | 850 | 1.01 | 0.91–1.11 | 0.917 | – | – | – |
| 4 | 814 | 0.95 | 0.86–1.04 | 0.3 | – | – | – |
| 5 | 851 | 1.03 | 0.94–1.13 | 0.563 | – | – | – |
| **Charlson Comorbidity Index** | | | | | | | |
| 0 | 813 | 1 | – | – | 1 | – | – |
| 1 | 284 | 1.58 | 1.38–1.81 | <0.001 | 1.11 | 0.97–1.27 | 0.128 |
| 2 | 3050 | 3.05 | 2.82–3.30 | <0.001 | 1.85 | 1.71–2.01 | <0.001 |
| **Diabetes** | | | | | | | |
| No | 3500 | 1 | – | – | 1 | – | – |
| Yes | 647 | 1.97 | 1.81–2.15 | <0.001 | 1.28 | 1.18–1.40 | <0.001 |
| **Operative indication** |  |  |  |  |  |  |  |
| Benign | 2821 | 1 | – | – | – | – | – |
| Malignant | 1326 | 1.07 | 1.00–1.14 | 0.055 | – | – | – |
| **Surgical access** |  |  |  |  |  |  |  |
| Open | 3396 | 1 | – | – | 1 | – | – |
| Minimally invasive | 151 | 0.27 | 0.23–0.32 | <0.001 | 0.35 | 0.30–0.41 | <0.001 |
| **B) Elective colorectal resections** | | | | | | | |
| **Covariate** | **N Dead (90 days)** | **Unadj. HR** | **95% CI** | ***P*-value** | **Adj. HR** | **95% CI** | ***P*-value** |
| **Sex** | | | | | | | |
| Male | 955 | 1 | – | – | 1 | – | – |
| Female | 564 | 0.6 | 0.54–0.67 | <0.001 | 0.6 | 0.54–0.67 | <0.001 |
| **Age (years)** |  |  |  |  |  |  |  |
| 16-50 | 67 | 1 | – | – | 1 | – | – |
| 51-60 | 111 | 2.23 | 1.65–3.02 | <0.001 | 1.9 | 1.40–2.58 | <0.001 |
| 61-70 | 331 | 4.25 | 3.27–5.53 | <0.001 | 3.55 | 2.71–4.65 | <0.001 |
| 71-80 | 555 | 7.03 | 5.46–9.06 | <0.001 | 5.86 | 4.50–7.63 | <0.001 |
| >80 | 455 | 12.62 | 9.77–16.32 | <0.001 | 10.85 | 8.30–14.18 | <0.001 |
| **Index of multiple deprivation quintile** | | | | | | | |
| 1 | 323 | 1 | – | – | – | – | – |
| 2 | 309 | 1.02 | 0.87–1.19 | 0.796 | – | – | – |
| 3 | 308 | 1.09 | 0.93–1.28 | 0.27 | – | – | – |
| 4 | 328 | 1.27 | 1.09–1.48 | 0.002 | – | – | – |
| 5 | 247 | 1.12 | 0.95–1.32 | 0.194 | – | – | – |
| **Charlson Comorbidity Index** | | | | | | | |
| 0 | 115 | 1 | – | – | 1 | – | – |
| 1 | 94 | 2 | 1.53–2.63 | <0.001 | 1.36 | 1.03–1.79 | 0.032 |
| 2 | 1310 | 3.04 | 2.52 ti 3.69 | <0.001 | 1.8 | 1.47–2.20 | <0.001 |
| **Diabetes** | | | | | | | |
| No | 1248 | 1 | – | – | 1 | – | – |
| Yes | 271 | 1.79 | 1.57–2.05 | <0.001 | 1.23 | 1.07–1.40 | 0.003 |
| **Operative indication** | | | | | | | |
| Benign | 414 | 1 |  |  |  |  |  |
| Malignant | 1105 | 1.34 | 1.19–1.50 | <0.001 | 0.71 | 0.63–0.81 | <0.001 |
| **Surgical access** | | | | | | | |
| Open | 1099 | 1 | – | – | 1 | – | – |
| Minimally invasive | 420 | 0.35 | 0.32–0.40 | <0.001 | 0.38 | 0.34–0.43 | <0.001 |

**Supplementary Table S4: Comparison of Accelerated Failure Time (AFT) models**

|  | **Distribution** | | | | |
| --- | --- | --- | --- | --- | --- |
| **Parameter** | Exponential (AFT) | Weibull (AFT) | Lognormal | Loglogistic | Generalised gamma |
| Log likelihood | -107714.93 | -106791.46 | -83561.984 | -81516.452 | NA |
| AIC | 215463.9 | 213618.9 | 167160 | 163068.9 | NA |

AIC= Akaike information criterion

**Supplementary Table S5: Effect of insulin use on 90-day mortality. CI, confidence interval; HR, hazard ratio. Cox regression model for 90-day mortality with use of insulin as the exposure of interest. Deprivation was included in the final model based on likelihood ratio test results (*P*<0.001).**

|  | | | | | | | | | | |
| --- | --- | --- | --- | --- | --- | --- | --- | --- | --- | --- |
| **Covariate** | **Person-years at risk** | **N Dead (90 days)** | **90-day Mortality Rate per 1000-person years** | **95% CI** | **Unadjusted HR** | **95% CI** | ***P*-value** | **Adjusted HR** | **95% CI** | ***P*-value** |
| **Sex** | | | | | | | | | | |
| Male | 1.3E+04 | 2891 | 231.36 | 222.98–239.84 | 1 | – | – | – | – | – |
| Female | 1.3E+04 | 2834 | 224.52 | 216.40–232.94 | 0.98 | 0.93–1.03 | 0.483 | – | – | – |
| **Age (years)** | | | | | | | | | | |
| 16-50 | 6.2E+03 | 370 | 59.35 | 53.60–65.71 | 1 | – | – | 1 | – | – |
| 51-60 | 4.0E+03 | 539 | 133.94 | 123.09–145.73 | 2.25 | 1.97–2.58 | <0.001 | 2.36 | 2.06–2.70 | <0.001 |
| 61-70 | 5.9E+03 | 1182 | 200.70 | 189.57–212.47 | 3.36 | 2.99–3.78 | <0.001 | 3.66 | 3.25–4.14 | <0.001 |
| 71-80 | 6.0E+03 | 1825 | 304.08 | 290.45–318.36 | 5.08 | 4.54–5.69 | <0.001 | 5.25 | 4.67–5.90 | <0.001 |
| >80 | 3.0E+03 | 1809 | 608.33 | 580.93–637.02 | 9.99 | 8.93–11.12 | <0.001 | 8.82 | 7.84–9.93 | <0.001 |
| **Index of multiple deprivation quintile** | | | | | | | | | | |
| 1 | 5.6E+03 | 1186 | 211.36 | 199.66–223.73 | 1 | – | – | 1 | – | – |
| 2 | 5.3E+03 | 1100 | 206.21 | 194.38–218.77 | 0.97 | 0.89–1.05 | 0.446 | 0.99 | 0.91–1.07 | 0.764 |
| 3 | 5.1E+03 | 1170 | 230.25 | 217.43–243.83 | 1.09 | 1.00–1.18 | 0.038 | 1.08 | 1.00–1.17 | 0.064 |
| 4 | 4.8E+03 | 1149 | 240.03 | 226.55–254.32 | 1.14 | 1.05–1.23 | 0.002 | 1.18 | 1.09–1.28 | <0.001 |
| 5 | 4.3E+03 | 1108 | 258.95 | 244.14–274.66 | 1.22 | 1.13–1.34 | <0.001 | 1.23 | 1.14–1.34 | <0.001 |
| **Charlson Comorbidity Index** | | | | | | | | | | |
| 0 | 6.6E+03 | 938 | 143.03 | 134.16–152.48 | 1 | – | – | 1 | – | – |
| 1 | 2.1E+03 | 382 | 183.84 | 166.30–203.23 | 1.28 | 1.13–1.44 | <0.001 | 1.17 | 1.04–1.32 | 0.011 |
| 2 | 1.6E+04 | 4405 | 267.17 | 259.39–275.17 | 1.84 | 1.72–1.98 | <0.001 | 1.79 | 1.66–1.93 | <0.001 |
| **Diabetes** | | | | | | | | | | |
| No | 2.3E+04 | 4798 | 212.27 | 206.34–218.36 | 1 | – | – | 1 | – | – |
| DM without insulin use | 2.0E+03 | 718 | 354.24 | 329.25–381.12 | 1.66 | 1.53–1.79 | <0.001 | 1.22 | 1.13–1.33 | <0.001 |
| DM with insulin use | 4.9E+02 | 209 | 423.76 | 370.03–485.29 | 1.95 | 1.70–2.25 | <0.001 | 1.51 | 1.31–1.74 | <0.001 |
| **Urgency of surgery** | | | | | | | | | | |
| Elective | 1.8E+04 | 1519 | 86.43 | 82.19–90.89 | 1 | – | – | 1 | – | – |
| Emergency | 7.4E+03 | 4147 | 556.88 | 540.19–574.09 | 6.25 | 5.89–6.63 | <0.001 | 4.83 | 4.52–5.16 | <0.001 |
| **Operative indication** | | | | | | | | | | |
| Benign | 1.1E+04 | 3235 | 292.95 | 283.03–303.22 | 1 | – | – | 1 | – | – |
| Malignant | 1.4E+04 | 2438 | 174.13 | 167.36–181.18 | 0.60 | 0.57–0.63 | <0.001 | 0.78 | 0.73–0.82 | <0.001 |
| **Surgical access** | | | | | | | | | | |
| Open | 1.5E+04 | 5149 | 342.48 | 333.25–351.96 | 1 | – | – | 1 | – | – |
| Minimally invasive | 1.0E+04 | 576 | 57.09 | 52.61–61.95 | 0.17 | 0.16–0.19 | <0.001 | 0.40 | 0.36–0.45 | <0.001 |

| **Covariate** | **Unadjusted HR** | **95% CI** | ***P-*value** | **Adjusted HR** | **95% CI** | ***P-*value** |
| --- | --- | --- | --- | --- | --- | --- |
| **Sex** | | | | | | |
| Male | 1 | – | – | 1 | – | – |
| Female | 0.96 | 0.94–0.99 | 0.009 | 0.95 | 0.92–0.98 | 0.003 |
| **Age (years)** | | | | | | |
| 0-50 | 1 | – | – | 1 | – | – |
| 51-60 | 0.92 | 0.88–0.96 | <0.001 | 0.92 | 0.88–0.97 | <0.001 |
| 61-70 | 0.82 | 0.79–0.86 | <0.001 | 0.83 | 0.80–0.87 | <0.001 |
| 71-80 | 0.83 | 0.80–0.86 | <0.001 | 0.83 | 0.80–0.87 | <0.001 |
| >80 | 0.84 | 0.80–0.88 | <0.001 | 0.83 | 0.79–0.87 | <0.001 |
| **Index of multiple deprivation score** | | | | | | |
| 1 | 1 | – | – | 1 | – | – |
| 2 | 1.04 | 1.00–1.08 | 0.08 | 1.02 | 0.98–1.07 | 0.214 |
| 3 | 1.05 | 1.01–1.10 | 0.027 | 1.02 | 0.98–1.06 | 0.393 |
| 4 | 1.15 | 1.10–1.20 | <0.001 | 1.10 | 1.05–1.15 | <0.001 |
| 5 | 1.28 | 1.23–1.34 | <0.001 | 1.19 | 1.14–1.25 | <0.001 |
| **Charlson Comorbidity Index** | | | | | | |
| 0 | 1 | – | – | 1 | – | – |
| 1 | 0.98 | 0.92–1.03 | 0.419 | 1.12 | 1.06–1.19 | <0.001 |
| 2 | 1.05 | 1.02–1.08 | 0.003 | 1.27 | 1.23–1.32 | <0.001 |
| **Diabetes** | | | | | | |
| No | 1 | – | – | 1 | – | – |
| DM without insulin use | 1.12 | 1.07–1.18 | <0.001 | 1.17 | 1.12–1.23 | <0.001 |
| DM with insulin use | 1.47 | 1.35–1.60 | <0.001 | 1.46 | 1.34–1.59 | <0.001 |
| **Urgency of surgery** | | | | | | |
| Elective | 1 | – | – | 1 | – | – |
| Emergency | 1.39 | 1.35–1.43 | <0.001 | 1.27 | 1.23–1.31 | <0.001 |
| **Operative indication** | | | | | | |
| Benign | 1 | – | – | 1 | – | – |
| Malignant | 0.74 | 0.71–0.76 | <0.001 | 0.84 | 0.81–0.86 | <0.001 |
| **Surgical access** | | | | | | |
| Open | 1 | – | – | 1 | – | – |
| Minimally invasive | 0.77 | 0.75–0.80 | <0.001 | 0.87 | 0.84–0.89 | <0.001 |

**Supplementary Table S6: Effect of Insulin use on 90-day readmission. CI, confidence interval; HR, hazard ratio. Cox regression model for 90-day readmission with use of insulin as exposure of interest. Deprivation was** **included in the final model based on likelihood ratio test results (*P*<0.001).**


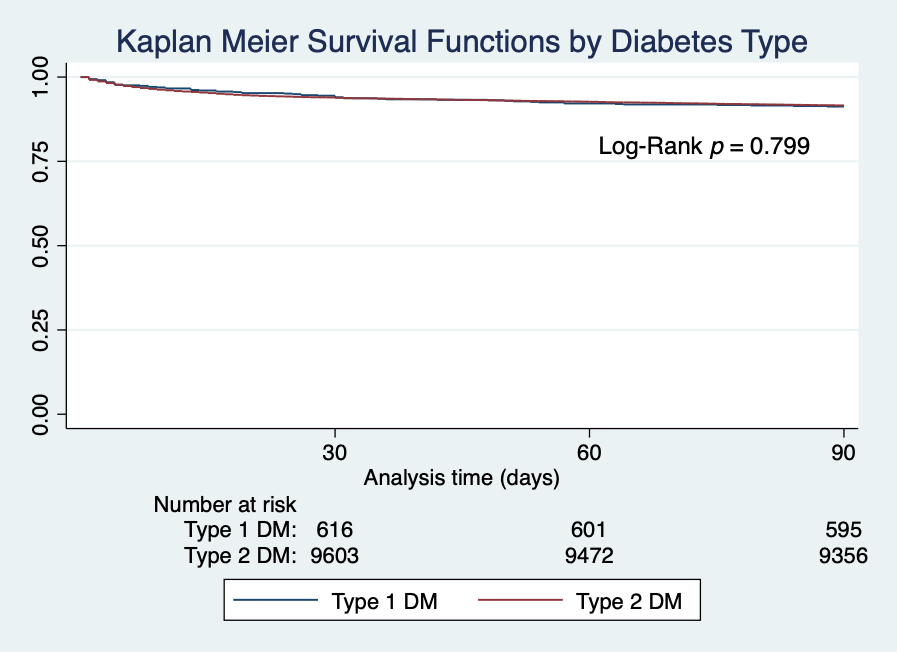


**Supplementary Figure S1:** Kaplan Meier survival functions stratified by diabetes type. DM = Diabetes Mellitus.


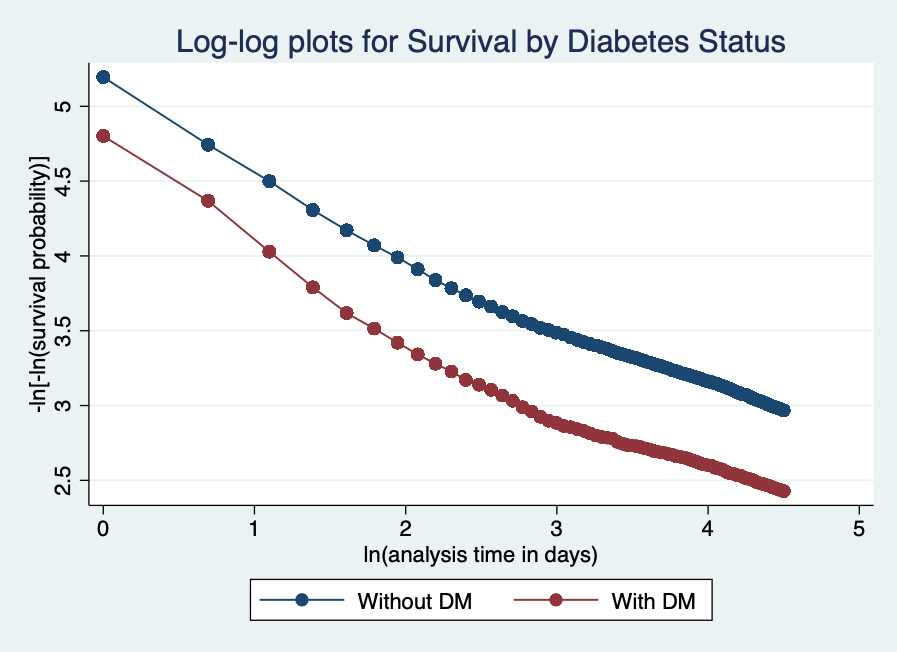


**Supplementary Figure S2:** Log-log plots for survival, stratified by diabetes status. DM = Diabetes Mellitus.


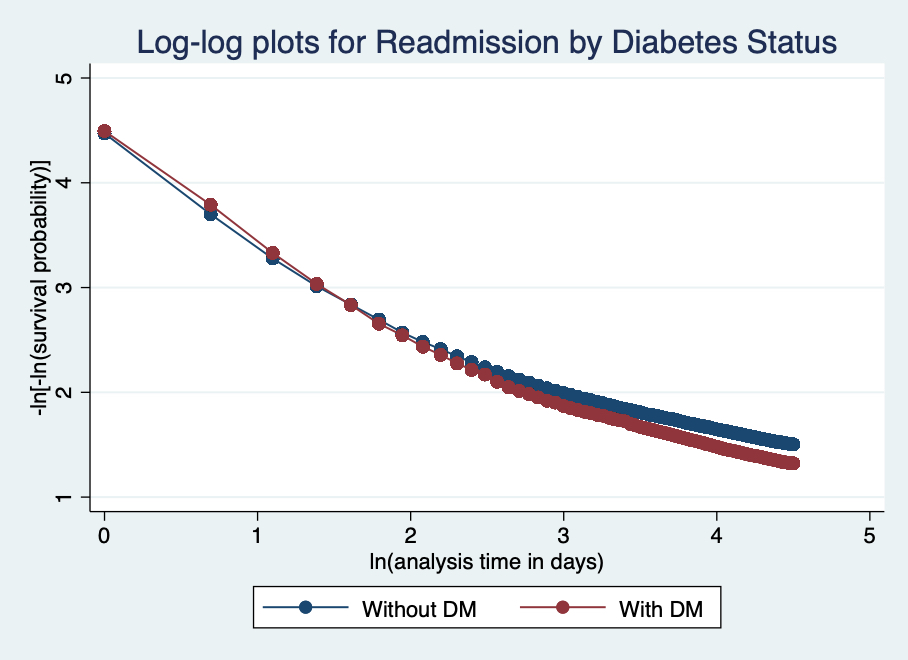


**Supplementary Figure S3:** Log-log plots for readmission, stratified by diabetes status. DM = Diabetes Mellitus.


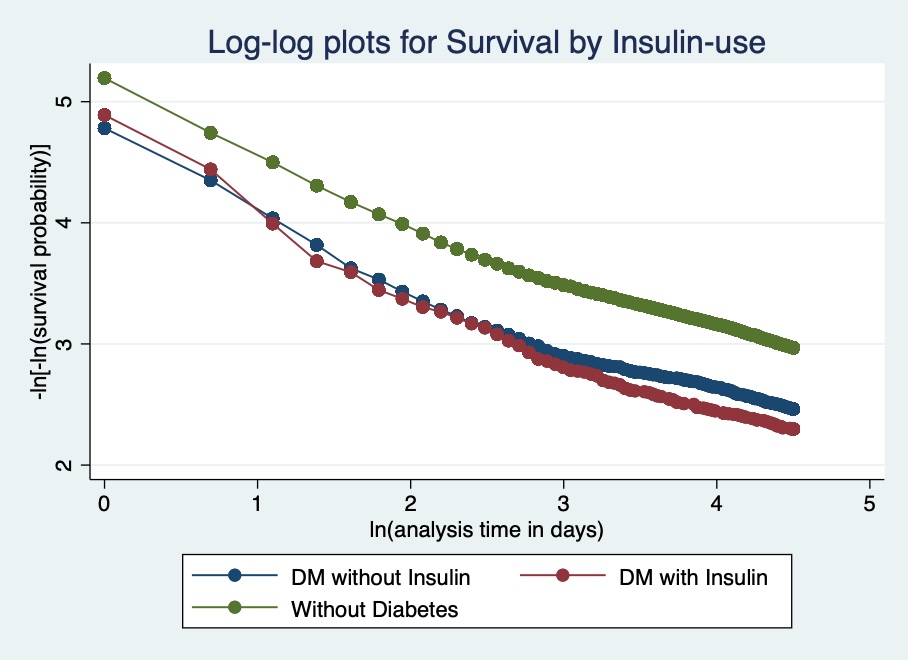


**Supplementary Figure S4:** Log-log plots for survival, stratified by use of insulin. DM = Diabetes Mellitus.


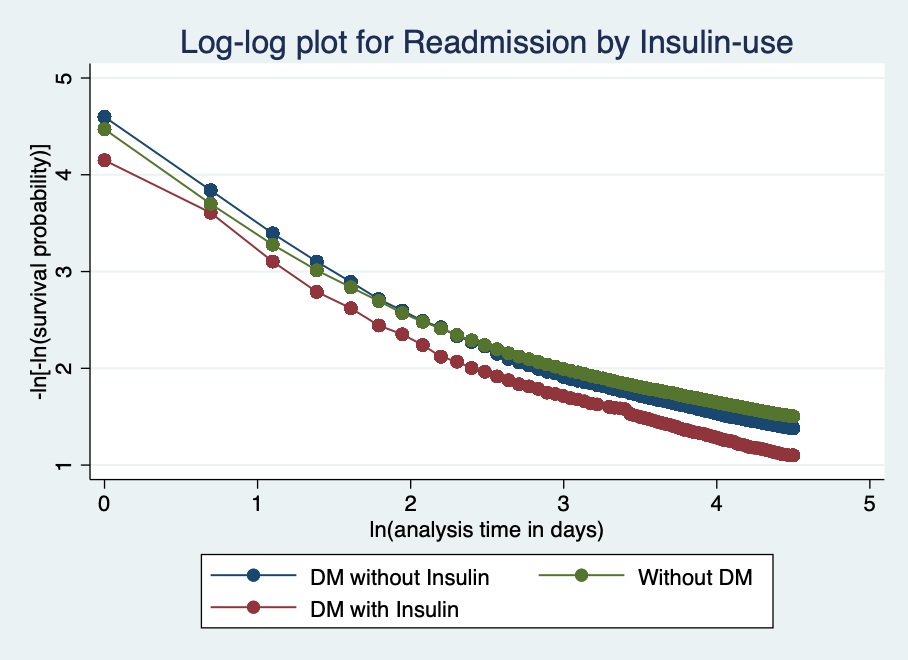


**Supplementary Figure S5:** Log-log plots for readmission, stratified by use of insulin. DM = Diabetes Mellitus.
